# Time-scale synchronisation of oscillatory responses can lead to non-monotonous R-tipping

**DOI:** 10.1038/s41598-023-28771-1

**Published:** 2023-02-06

**Authors:** Jan Swierczek-Jereczek, Alexander Robinson, Javier Blasco, Jorge Alvarez-Solas, Marisa Montoya

**Affiliations:** 1 Department Earth Science and Astrophysics, Complutense University of Madrid, Madrid, Spain; 2 Geosciences Institute, CSIC-UCM, Madrid, Spain; 3 Potsdam Institute for Climate Impact Research, Potsdam, Germany; 4 Free University of Brussels, Brussels, Belgium

**Keywords:** Applied mathematics, Climate sciences

## Abstract

Rate-induced tipping (R-tipping) describes the fact that, for multistable dynamic systems, an abrupt transition can take place not only because of the forcing magnitude, but also because of the forcing rate. In the present work, we demonstrate through the case study of a piecewise-linear oscillator (PLO), that increasing the rate of forcing can make the system tip in some cases but might also prevent it from tipping in others. This counterintuitive effect is further called non-monotonous R-tipping (NMRT) and has already been observed in recent studies. We show that, in the present case, the reason for NMRT is the peak synchronisation of oscillatory responses operating on different time scales. We further illustrate that NMRT can be observed even in the presence of additive white noise of intermediate amplitude. Finally, NMRT is also observed on a van-der-Pol oscillator with an unstable limit cycle, suggesting that this effect is not limited to systems with a discontinuous right-hand side such as the PLO. This insight might be highly valuable, as the current research on tipping elements is shifting from an equilibrium to a dynamic perspective while using models of increasing complexity, in which NMRT might be observed but hard to understand.

## Introduction

For a multistable, dynamic system, crossing a basin boundary leads to abrupt changes in the observed dynamics. This is commonly referred to as tipping and can occur through the presence of a bifurcation (B-tipping), a dynamic forcing displaying a rate (R-tipping), a forcing subject to noise (N-tipping) or a single perturbation (S-tipping). Here, “or” should not be understood as an exclusive statement, as different types of tipping might be combined - for instance, when a bifurcating system is subject to a stochastic forcing displaying a trend. Due to the complexity of models representing real-world tipping elements, former studies often focused on characterising bifurcation tipping (B-tipping)^1–4^. B-tipping relies on the arguably wrong, albeit conceptually very helpful view of an equilibrated system and recent studies have started to fill this gap by accounting for the effect of the forcing rate on conceptual^5–7^, as well as on higher-complexity models^8^.

Rate-induced tipping (R-tipping) might obey a monotonous logic in our intuition: the higher the forcing rate, the more likely the escape of the basin of attraction due to the dynamic nature of the forcing. For instance, in^5,9^, a forcing rate *R* is derived below which the system does not tip, whereas it tips if it is higher. Further work has investigated R-tipping in the context of systems displaying attractors rather than equilibria^10^. In this setting, the initial condition can be any point of the attractor and a family of initial conditions is therefore considered. For forcing rates that are low enough, all the associated trajectories are able to track the attractor. Beyond a certain value, only some of them are able to do so - a situation called *partial tipping*. Above a certain value of the rate, *total tipping* arises, i.e. none of the trajectories remain on the attractor. Here again, a monotonous vision of R-tipping is represented, albeit generalised beyond equilibria. In contrast, an example escaping this logic would be to successively obtain no tipping, partial tipping, total tipping and partial tipping again by increasing the rate.

Monotonicity with respect to the rate parameter has been proven not to be a universal property in^11^, where the intricate shape of the bifurcation branches lead to non-monotonous R-tipping (NMRT) with respect to the forcing rate. More precisely, for three different rates $0< r_{1}< r_{2} < r_{3}$, tipping can be observed for the intermediate value but not for the lower nor the higher ones. In essence, this means that increasing the rate might prevent R-tipping in some cases, which may appear counterintuitive at first. Another example of NMRT has been observed in an ecological model^7^ as well as in a chaotic system presenting non-monotonous tipping probabilities^12^. NMRT has also been observed in a comprehensive model of the Atlantic Meridional Overturning Circulation (AMOC)^8^, where chaos has been suggested to play an important role in the observed patterns. These four examples undermine R-tipping as a monotonous phenomenon with respect to the forcing rate - at least in the general sense. It is worth mentioning, that the effect of a non-monotonous forcing has been studied in e.g.^6,7^. In this context, obtaining non-monotonities in the resulting tipping behaviour might however appear less surprising. We emphasise that the present article only considers the the monotonous forcing case.

In the present work, we propose a mechanism for NMRT that does not rely on the structure of the bifurcation branches as in^11^, but rather on the oscillatory behaviour of the system. To illustrate this, we first introduce the model of a piecewise-linear oscillator (PLO). A set of experiments is driven, showing that the PLO is capable of displaying NMRT despite its low complexity (non-chaotic, deterministic, two-dimensional system displaying equilibrium points). By means of the superposition principle, we subsequently show that, in the present case, NMRT can be explained by the peak synchronisation of oscillatory responses displaying different time scales. As real-world forcing is often subject to stochasticity, we further investigate the NMRT behaviour in combination with noise-induced tipping (N-tipping). Finally, in order to study whether peak-synchronised NMRT is specific to the structure of the PLO, we study a van-der-Pol oscillator (VPO) with an unstable limit cycle. As NMRT can be observed here again, we show that the insights gained in the present work seem to be generalisable to oscillatory systems.

## Results

### Model of a simple piecewise-linear oscillator

**Figure 1:**
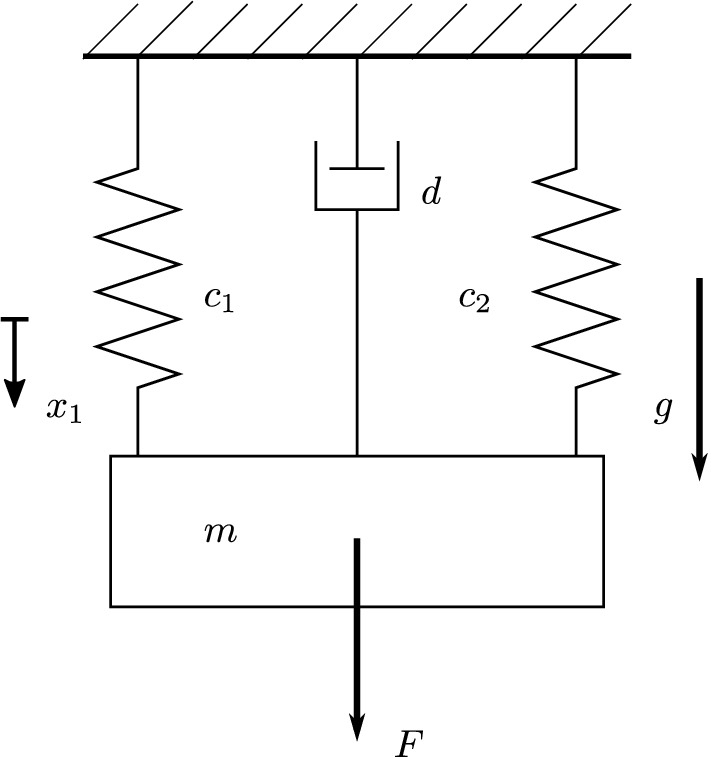
Mechanical sketch of the PLO.

As illustrated in Fig. 1, we introduce a point mass $m \in {\mathbb {R}}_{+} \setminus \left\{ 0 \right\}$ linked to a rigid wall by two springs and a damper, with constants $c_{1}, c_{2}, d \in {\mathbb {R}}_{+} \setminus \left\{ 0 \right\}$ respectively. We define the time $t \in {\mathbb {R}}_{+}$ and the downward pointing coordinate $x_{1} \in {\mathbb {R}}$ by setting $x_{1}=0 \, {\textrm{m}}$ to be the position of zero spring deformation. The mass is subject to the gravitational force and the external force $F(t) \in {\mathbb {R}}$. Note that through the point-mass assumption there is no rotational degree of freedom and the problem becomes one-dimensional because of the coaxiality of all forces involved. By introducing the velocity $x_{2}(t) = {\dot{x}}_{1}(t)$ and applying Newton’s second law of motion, we obtain following governing equation:

1 $$\begin{aligned} \dfrac{{\textrm{d}}x}{{\textrm{d}}t}&= \dfrac{{\textrm{d}}}{{\textrm{d}}t} \begin{bmatrix} &{} x_{1} &{} \\ &{} x_{2} &{} \end{bmatrix} = \begin{bmatrix} &{} 0 &{} 1 &{} \\ &{} - {c}/{m} &{} - {d}/{m} &{} \end{bmatrix} \begin{bmatrix} &{} x_{1} &{} \\ &{} x_{2} &{} \end{bmatrix} + \dfrac{1}{m} \begin{bmatrix} &{} 0 &{} \\ &{} mg + F(t) &{} \end{bmatrix}. \end{aligned}$$

The numerical values used for this case study are summarised in Table 1 of the supplementary material (low-frequency case). In particular, it should be noted that *m*, *d* and $c_{1}$, $c_{2}$ and are chosen such that the system presents oscillatory behaviour with damping ratio $D = d \, \left( 2 \, \sqrt{m \, c } \right) ^{-1} \simeq 0.32$ and total stiffness $c = c_{1} + c_{2}$.

So far, the system is linear and therefore cannot display tipping. To embed this possibility in the model while keeping its complexity low, we define $c_{2}$ to depend on the history of the system at fixed but arbitrary time ${\tilde{t}}$:

2 $$\begin{aligned} c_{2}({\tilde{t}}) = \left\{ \begin{array}{ll} c_{2}, &{}\quad \forall t \le {\tilde{t}}: \, \max \, x_{1}(t) \le x_{T}. \\ 0, \, &{}\quad \forall t \le {\tilde{t}}: \, \max \, x_{1}(t) > x_{T}. \\ \end{array} \right. \end{aligned}$$

Physically speaking, this can be understood as a sudden rupture of the spring at $x_{1} = x_{T}$ that cannot be recovered. In other words, one of the negative feedbacks abruptly disappears, leading to a different equilibrium. This resembles the ice-albedo parametrisation often used in former conceptual energy balance models^13^. We emphasise that, in order to ease the later-driven analysis, no plasticity model has been used to represent the snapping of spring 2. The fact that a bifurcation is introduced in form of a rupture hysteresis becomes obvious when studying the equilibrium point ${\tilde{x}}$ of Eq. (1), associated with the constant forcing $F(t) = {\tilde{F}} \in {\mathbb {R}}$ and ${\dot{x}}_{1} = \ddot{x}_{2} \equiv 0$:

3 $$\begin{aligned} {\tilde{x}}({\tilde{F}})= \left\{ \begin{array}{ll} {\tilde{x}}_{-}({\tilde{F}}), &{}\quad {\text {if spring 2 is not broken}} \\ {\tilde{x}}_{+}({\tilde{F}}), &{}\quad {\text {if spring 2 is broken}} \end{array} \right. , \end{aligned}$$

4 $$\begin{aligned} {\mathrm {with:}} \qquad {\tilde{x}}_{-}({\tilde{F}})= \dfrac{{\tilde{F}} + mg}{c_{1} + c_{2}}, \qquad {\tilde{x}}_{+}({\tilde{F}}) = \dfrac{{\tilde{F}} + mg}{c_{1}}. \end{aligned}$$

The resulting bifurcation diagram and the autonomous phase space are provided in Fig. 1 of the supplementary material.

### Non-monotonous R-tipping

To study R-tipping in complex models, a possible approach is to apply various saturated ramps^6,8^ with different slopes *a* and saturation levels $F_{\max }$. Together, these two values constitute the parameter space of the forcing. The time at which the saturated ramp reaches its maximum is given by ${\hat{t}} = F_{\max } \cdot a^{-1}$ and *F*(*t*) can therefore be expressed by:

5 $$\begin{aligned} F(t) = \left\{ \begin{array}{ll} a t &{}\quad \forall t < {\hat{t}} \\ F_{\max } &{}\quad \forall t \ge {\hat{t}} \end{array} \right. . \end{aligned}$$

This forcing is applied on the PLO for 50 linearly sampled values of $F_{\max } \in [35, 52] \, {\textrm{N}}$ and 50 logarithmically sampled values of $a \in [10^{-2}, 10^{3}] {\mathrm {N \, s}}^{-1}$. We set the initial state of spring 2 to be unbroken and the initial conditions to be equilibrated with the gravitational force:

6 $$\begin{aligned} x(t=0) = \begin{bmatrix}&{\tilde{x}}_{-}({\tilde{F}}=0)&0&\end{bmatrix}^{\textrm{T}}. \end{aligned}$$

The joint consideration of Eqs. (1), (5) and (6) delivers an initial value problem (IVP) for each sampled pair $( F_{\max }, a )$, which is solved by applying the 5th-order Tsitouras-method^14^. The time span is chosen to be large enough to ensure that the system is at equilibrium with $F_{\max }$ at end time $t_{e}$. The end position $x_{1}(t=t_{e})$ gives unambiguous information on whether the system has tipped ($x_{1}(t=t_{e}) = {\tilde{x}}_{+}(F_{\max }) > x_{T}$) or not ($x_{1}(t=t_{e}) = {\tilde{x}}_{-}(F_{\max }) < x_{T}$).

**Figure 2:**
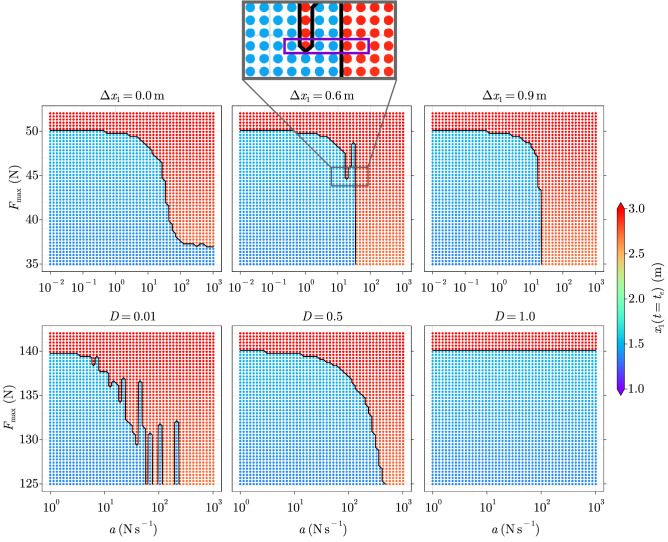
Deterministic tipping pattern of the PLO in the ramp-parameter space for (top row) $\Delta x_{1} = \lbrace 0, 0.6, 0.9 \rbrace \, {\textrm{m}}$ and $D \simeq 0.32 \, {\textrm{m}}$ and (bottom row) $D = \lbrace 0.01, 0.5, 1 \rbrace$ and $\Delta x_1 = 0.6 \, {\textrm{m}}$. In the bottom row, the eigenfrequency of the PLO is increased by reducing the mass as specified in Table 1 of the supplementary material. The black curve represents the separation function $F_{\textrm{min,tip}}(a)$. The time series associated with the points in the violet rectangle of the top-centre panel are visualised in Fig. 3.

The tipping behaviour of the solution in the sampled ramp-parameter space is visualised in the top-left panel of Fig. 2. The end position displays a largely binary behaviour which can be expected from the snapping behaviour of spring 2. We define $F_{{\textrm{min,tip}}}(a)$ to be the minimum forcing amplitude required for the system to tip for a given slope *a*. This function separates the region containing the experiments that do not lead to tipping from those leading to tipping and is therefore called the *separation function*, showing the following asymptotic behaviour:

7 $$\begin{aligned} \underset{a \rightarrow 0}{\lim }\ F_{{\textrm{min,tip}}}(a) = F_{T} = 50 \, {{\textrm{N}}}, \qquad \underset{a \rightarrow \infty }{\lim } F_{{\textrm{min,tip}}}(a) \simeq 37 \, {{\textrm{N}}}. \end{aligned}$$

For low slopes $a < 2 \cdot 10^{-1} \, {{\mathrm {N \, s}}}^{-1}$, a ramp forcing allows the system to track equilibrium and the separation function is close to the bifurcation point. It can thus be used to generate the hysteresis curve of a system, as it was done in^1,3^ for tipping elements of the Earth system. For high slopes $a > 10^{2} \, {{\mathrm {N \, s}}}^{-1}$, tipping occurs for much lower values of the saturation level which is the manifestation of R-tipping. From there on, increasing the rate has barely any influence, as the low-pass nature of the PLO seems to cut out the additional high-frequency content of the forcing signal. Effectively, this means that the forcing response is close to the step response for $a > 10^{2} \, {{\mathrm {N \, s}}}^{-1}$. Between both asymptotes, the separation function is monotonously decreasing with the slope, which might appear as an intuitive result. Nonetheless, it is possible to find cases that deviate from this expected behaviour. To demonstrate this, we let the initial condition depend on an initial perturbation $\Delta x_{1}$:

8 $$\begin{aligned} x(t=0) = \begin{bmatrix}&{\tilde{x}}_{-}(0) - \Delta x_{1}&0&\end{bmatrix}^{\textrm{T}}. \end{aligned}$$

This corresponds to the introduction of a second time scale in the forcing and therefore in the solution since, in addition to the ramp response, a transient response to the initial perturbation is now also present. We repeat the solving procedure of the IVPs for $\Delta x_{1} = \left\{ 0.6, 0.9 \right\} \, {\textrm{m}}$. The resulting R-tipping patterns are shown in the top-centre and top-right panels of Fig. 2. For $\Delta x_{1} = 0.6 \, {\textrm{m}}$, a non-monotonous separation $F_{{\textrm{min,tip}}}(a)$ is clearly displayed, justifying the choice of NMRT as a terminology. In particular, this can be seen by the alternating pattern shown in the purple rectangle: at constant saturation level and for increasing rate, the experiments successively display no-tipping, tipping, no-tipping and finally tipping again.

The possibility of such behaviour has already been suggested in^11^ as a consequence of intricate shapes of bifurcation branches of the studied system. However, the branches have a simple topology in the present case and this explanation does not apply. This challenges the understanding of R-tipping that has been gained until now and may sound counter-intuitive. To understand why this happens, we take advantage of the fact that the system is linear until the spring snaps. In particular, we split the solution *x*(*t*) into two parts by using the superposition principle. We define:

9 $$\begin{aligned} x(t) = x^{\textrm{F}}(t) + x^{g}(t), \end{aligned}$$

with $x^{F}(t), \, x^{g}(t) \in {\mathbb {R}}^2$ being the respective solutions of following IVPs:

10 $$\begin{aligned}&\text {Find}\, x^{F}(t) \text { such that:}&\left\{ \begin{array}{ll} \dfrac{{{\textrm{d}}}}{{{\textrm{d}}}t} \begin{bmatrix} x_{1}^{F} \\ x_{2}^{F} \end{bmatrix} = \begin{bmatrix} &{} 0 &{} 1 &{} \\ &{} - {c}/{m} &{} - {d}/{m} &{} \end{bmatrix} \begin{bmatrix} x_{1}^{F} \\ x_{2}^{F} \end{bmatrix} + \dfrac{1}{m} \begin{bmatrix} 0 \\ F(t) \end{bmatrix} \\ \ \\ x^{F}(t=0) = \begin{bmatrix} &{} 0 &{} 0 &{} \end{bmatrix}^{\textrm{T}} \end{array} \right. \end{aligned}$$

11 $$\begin{aligned}&\text {Find}\, x^{g}(t) \text { such that:}&\left\{ \begin{array}{ll} \dfrac{{\textrm{d}}}{{\textrm{d}}t} \begin{bmatrix} x_{1}^{g} \\ x_{2}^{g} \end{bmatrix} = \begin{bmatrix} &{} 0 &{} 1 &{} \\ &{} - {c}/{m} &{} - {d}/{m} &{} \end{bmatrix} \begin{bmatrix} x_{1}^{g} \\ x_{2}^{g} \end{bmatrix} + \dfrac{1}{m} \begin{bmatrix} 0 \\ mg \end{bmatrix} \\ \ \\ x^{g}(t=0) = \begin{bmatrix} &{} {\tilde{x}}_{-}(0) - \Delta x_{1} &{} 0 &{} \end{bmatrix}^{\textrm{T}} \end{array} \right. \end{aligned}$$

**Figure 3:**
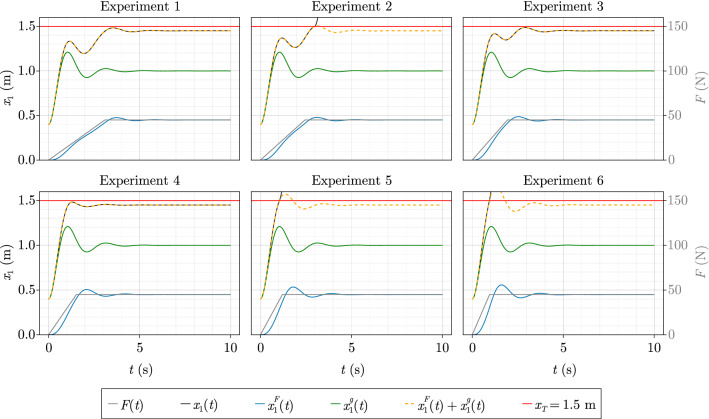
Superposed positions of the experiments in the violet rectangle of Fig. 2. Whenever the superposed position yields a position $x_{1}^{g} + x_{1}^{F}$ that is larger than the snapping displacement $x_{T}$, the full nonlinear position $x_{1}(t)$ displays tipping.

While $x^{F}(t)$ represents the response to the saturated ramp, $x^{g}(t)$ represents the response to the initial perturbation. Neither of the IVPs include the nonlinearity, i.e. their sum only corresponds to the full nonlinear solution *x*(*t*) until the spring snaps. Despite this difference, tipping can still be assessed whenever the superposed position $x_{1}^{F}(t) + x_{1}^{g}(t)$ is greater than the snapping threshold $x_{T}$. The time series associated with the points enclosed by the violet rectangle in the top-centre panel of Fig. 2 are displayed in Fig. 3 and give insights on why experiments 2, 5 and 6 lead to tipping, while 1, 3 and 4 do not, although the forcing rate increases as the experiment number increases:

- Experiment 1 does not lead to tipping because the first crest of $x^{F}_{1}(t)$ (blue) is roughly in phase with the second trough of $x^{g}_{1}(t)$ (green). Thus, the maximum of the superposed position (orange dashed) is smaller than the snapping threshold $x_{T} = 1.5 \, {\textrm{m}}$ (red). In experiments 3 and 4, a similar synchronisation happens, however with the first trough of $x^{g}_{1}(t)$.
- Experiment 2 leads to tipping because the first crest of $x^{F}_{1}(t)$ coincides with the second crest of $x^{g}_{1}(t)$ and thus causes the superposed position to cross $x_{T}$.
- Finally, in experiments 5 and 6, tipping occurs because $x^{F}_{1}(t)$ has already reached a value that is high enough when $x^{g}_{1}(t)$ reaches its first crest.

Thanks to the superposition principle, it becomes obvious that NMRT is due to the in-phase/out-of-phase behaviour of the responses. An important insight is that this peak-synchronisation can only happen if the system is capable of oscillating. In particular, this behaviour should not be possible for nonlinear systems of dimension $n=1$, as they are incapable of oscillation^15^.

As can be seen in the top-right panel of Fig. 2, this non-monotonous behaviour is not always present. This is due to the fact that for large $\Delta x_{1}$, the magnitude of $x^{g}$ becomes dominant. Conversely, for low values of $\Delta x _{1}$, the dominating magnitude is that of the saturated ramp, as depicted in the top-left panel of Fig. 2. Non-monotonous R-tipping is therefore not only a matter of time synchronisation, but also of comparable magnitudes in the various responses.

Based on this understanding, one can predict that for a PLO with higher damping $0.32<D<1$, the synchronisation effect is less likely to occur, whereas R-tipping simply cannot occur if the system is overdamped $D \ge 1$, i.e. incapable of oscillating. Conversely, for a higher eigenfrequency of the PLO and lower damping, the synchronisation events are more likely to happen, giving rise to a denser alternation pattern between tipped and non-tipped regions. To test these hypotheses, we run a set of experiments with fixed initial perturbation $\Delta x_{1} = 0.6 \, {\textrm{m}}$, different values of the damping ratio $D \in \lbrace 0.01, 0.5, 1 \rbrace$ and a lower mass (c.f. high-frequency case of Table 1 of supplementary material). The results are represented in the bottom row of Fig. 2. The first and second hypotheses are respectively confirmed by the centre and right panels, whereas the left panel confirms the third one.

The last observation shows that many alternating fringes can arise in the tipping pattern, which is not possible in the case of NMRT arising from the bifurcation topology as introduced in^11^. Therefore, although NMRT is observed in both cases, the underlying reasons for it and its resulting pattern are different. In particular, the present finding suggests that for an oscillatory system with fixed saturation level of the forcing, a rate $R_{1}$ can be found such that $0< r < R_{1}$ leads to tracking, while a rate $R_{2}$ can be found such that $r > R_{2}$ systematically leads to tipping. For $R_{1}< r < R_{2}$, tipping however depends on whether peak synchronisation arises or not. This behaviour is similar to the one of partial tipping as introduced in^10^, where a complex oscillator is not only externally forced but also perturbed in its initial conditions. Although a link might be present between both studies, we stress that in the present one the system displays equilibrium points instead of attractors.

### Stochastic non-monotonous R-tipping

The forcing of many real-world systems is often subject to noise, which sometimes stands for effects that are not captured by the model. For instance one can think of the atmospheric temperature forcing being subject to meteorological fluctuations, which can be simplified as an additive source of noise. To understand its effect on the present case study, we propose to add white noise of variance $\sigma ^2$ to the forcing, resulting in following SDE:

12 $$\begin{aligned} {\textrm{d}}x= & {} \left( \begin{bmatrix} &{} 0 &{} 1 &{} \\ &{} - {c}/{m} &{} - {d}/{m} &{} \end{bmatrix} \begin{bmatrix} &{} x_{1} &{} \\ &{} x_{2} &{} \end{bmatrix} + \dfrac{1}{m} \begin{bmatrix} &{} 0 &{} \\ &{} mg + F(t) &{} \end{bmatrix} \right) {\textrm{d}}t + \dfrac{1}{m} \begin{bmatrix} &{} 0 &{} \\ &{} \sigma &{} \end{bmatrix} dW, \end{aligned}$$

with *dW* the standard Brownian motion. In the forcing-parameter space, we reduce the domain of interest to $(F_{max}, a) \in [40, 50] \, {\textrm{N}} \times [1, 100] \, {\mathrm {N \, s}}^{-1}$, i.e. where the non-monotonous effect is observed in the top row of Fig. 2. Equation (12) is integrated by using an Euler–Maruyama scheme, with time step and end time fixed to $\Delta t = 0.01 \, {\textrm{s}}$ and $t_{e} = 60 \, {\textrm{s}}$, respectively. To study the influence of the introduced stochasticity, we run Monte-Carlo simulations with $N=100$ realisations per experiment with fixed $(F_{\max }, a)$. We repeat this for various values of the noise standard-deviation $\sigma = \left\{ 0.3, 1, 2 \right\} \, {\textrm{N}}$, thus representing cases from relatively low to high noise regimes, as the forcing required for a bifurcation to occur is $F = 50 \, {\textrm{N}}$. We define $n(F_{max}, a)$ as the number of realisations that tipped for a given experiment. This allows to compute the empirical tipping probability ${\hat{P}}_{\textrm{tip}}$, approximating the actual tipping probability $P_{\textrm{tip}}$ associated with a combination of the forcing parameters:

13 $$\begin{aligned} {\hat{P}}_{\textrm{tip}}(F_{\max }, a) = \left. {\hat{P}} \left( x_{e} > 1.5 \, {\textrm{m}} \right) \right| _{ F_{max}, a} = \dfrac{n(F_{max}, a)}{N}. \end{aligned}$$

**Figure 4:**
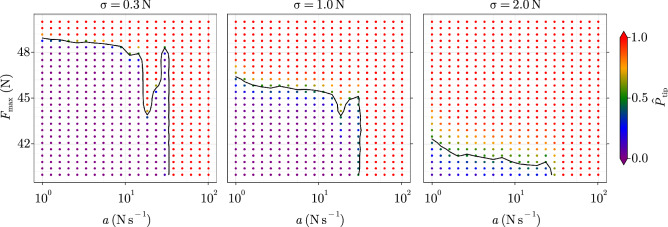
Probabilistic tipping pattern of the PLO in the ramp-parameter space for various noise amplitudes $\sigma$. The initial perturbation is fixed to $\Delta x_1 = 0.6 \, {\textrm{m}}$ and the damping ratio to $D \simeq 0.32 \, {\textrm{m}}$. The black curve represents the separation function $F_{\textrm{min,tip}}(a)$ between ${\hat{P}}_{\textrm{tip}} < 0.5$ and ${\hat{P}}_{\textrm{tip}} > 0.5$.

The resulting statistical tipping pattern is displayed in Fig. 4. The separation function is now redefined in the stochastic context as the function separating points with a tipping probability of less than 0.5 from those of more than 0.5. For the lower noise regime $\sigma = 0.3 \, {\textrm{N}}$, the shape of the associated curve is not qualitatively altered compared to the previously observed deterministic pattern. However, a single realisation might alter the separation curve, as isolated points can arise. This can be seen in Fig. 2 of the supplementary material, where some tipped experiments are exclusively surrounded by non-tipped experiments and vice-versa. Therefore the shape of the separation function is only preserved for $N \gg 1$. For the intermediate noise regime $\sigma \simeq 1 \, {\textrm{N}}$ displayed in Fig. 4, NMRT can still be observed, although its manifestation largely vanishes. For the higher noise regime $\sigma = 2 \, {\textrm{N}}$, the probabilistic separation function becomes monotonous, because the noise becomes dominant compared to the synchronisation effects of transient responses. NMRT can therefore be “hidden” by noise if the magnitude of the latter is large enough. As expected, it can be observed that the safe-operating space shrinks with increasing noise variance. More precisely, it appears that the tipping asymptote for low slopes is shifted towards smaller values of $F_{\max }$.

### Non-monotonous R-tipping of unstable Van-der-Pol oscillator

To investigate whether peak-synchronised NMRT can arise in a system with continuously differentiable right-hand side, we now introduce the unstable Van-der-Pol oscillator (VPO) governed by:

14 $$\begin{aligned} \dfrac{{\textrm{d}}}{{\textrm{d}}t} \begin{bmatrix} x_{1} \\ x_{2} \end{bmatrix} = \begin{bmatrix} - x_{2} \\ x_{1} - \mu \left( 1 - x_{1}^{2}\right) {x}_{2} \end{bmatrix} - \begin{bmatrix} 0 \\ F(t) \end{bmatrix}. \end{aligned}$$

All the variables and parameters are unitless. The system is forced by a time-dependent input *F*(*t*). For $F(t) = 0$, it displays a stable equilibrium point ${\tilde{x}} = 0 \in {\mathbb {R}}^2$ surrounded by an unstable limit cycle. The degree of nonlinearity of the system as well as the shape of the limit-cycle are determined by the parameter $\mu$. More generally, for a constant forcing $F(t) = F > 0$, an equilibrium point arises at ${\tilde{x}} = \left[ F \quad 0 \right] ^{\textrm{T}}$. Linearising around this point allows us to study its stability regime, which results in being stable for $|F|<1$ and unstable for $|F|>1$. At $F=1$ the limit cycle collapses into the equilibrium point, giving rise to a bifurcation.

**Figure 5:**
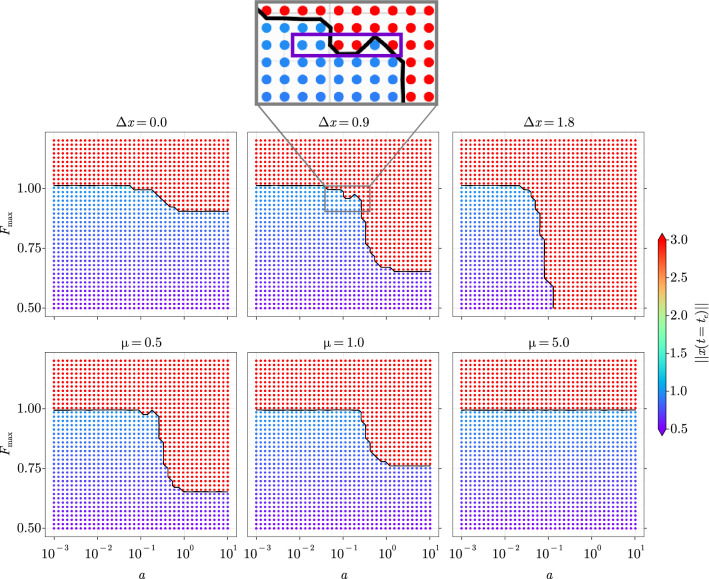
Tipping pattern of the VPO in the ramp-parameter space for (top row) $\Delta x_1 \in \lbrace 0.0, 0.9, 1.8 \rbrace$ and $\mu = 0.3$ and (bottom row) $\mu \in \lbrace 0.5, 1.0, 5.0 \rbrace$ and $\Delta x = 1$. The black curve represents the numerical approximation of the separation function $F_{\textrm{min,tip}}(a)$. The time series associated with the points enclosed by the purple rectangle in the top-centre panel are shown in Fig. 3 of the supplementary material.

Driving similar experiments to section, the VPO is forced with ramps displaying saturation levels and slopes sampled such that $F_{\max } \in [0.5, 1.2]$ and $a \in [10^{-3}, 10^{1}]$. The initial state $x_{1}$ is perturbed by $\Delta x_1 \in \lbrace 0.0, 0.9, 1.8 \rbrace$ in order to introduce an additional time scale to the one of the forcing, while the damping $\mu = 0.3$ is held constant. The resulting R-tipping pattern is displayed in the top row of Fig. 5 and shows that, as observed on the PLO, three main cases arise with increasing perturbation: (1) for a zero perturbation, NMRT does not arise while (2) it arises for perturbations of intermediate magnitude and (3) disappears whenever its magnitude is high enough for $x^{g}(t)$ to dominate the overall response.

Another set of experiments is performed by fixing the initial perturbation $\Delta x_1 = 1.0$ and varying the damping parameter $\mu = \lbrace 0.5, 1, 2 \rbrace$. As for the PLO, we observe three main cases with increasing damping: (1) the R-tipping pattern displays non-monotonous features, (2) NMRT is prohibited by the larger damping, however R-tipping itself is still possible and (3) R-tipping is absent due to the large damping.

NMRT is therefore not specific to piecewise-linear systems and can arise in non-chaotic ODEs with smooth right-hand sides. As was shown for the PLO, the reason for this effect to arise is suspected to be the synchronisation of two different oscillatory responses. Because the VPO is not piecewise-linear, the superposition principle cannot be applied. Nonetheless, one can still plot the sub-solutions in order to display how the system would react to separate forcings. As can be observed in Fig. 3 of the supplementary material, experiments displaying tipping also show a crest-crest synchronisation of sub-solutions, whereas non-tipped ones show a crest-trough one. This does not provide a rigorous analysis but delivers an intuition for why NMRT is happening. A formal proof of this remains as future work.

## Discussion

The presence of multiple forcing time scales is common in complex systems: an example of this is the time scale of atmospheric vs. oceanic warming, both drivers of the evolution of many Earth system components. From a modelling perspective, these quantities can be viewed as states if an energy balance with radiative forcing is included. Because the definition of a variable as state or forcing depends on how the limits of the models are set, a single forcing time scale can give rise to multiple internal time scales.

Moreover, one can think of examples where, as in the case of the PLO, the initial condition is not at equilibrium. This is the case for some high-resolution models of the AMOC, where control runs display a sustained oscillation as response^8,16,17^. Although the NMRT patterns observed in^8^ are similar to those of the present study (Fig. 2, bottom-left panel), it remains unclear whether peak-synchronisation is involved and to which extent it is connected to partial tipping.

Peak-synchronised NMRT gives rise to regions of the forcing-parameter space, where unexpected fringes of non-tipped experiments can be observed, as in the top-centre and more prominently in the bottom-left panels of Fig. 2. If tipping is to be avoided, these regions could still be arguably classified as “dangerous” for three reasons:

- Models present structural and parametric uncertainties. If these uncertainties are not propagated to the solution, an alternating tipping pattern is likely to be shifted in simulation compared to reality.
- Real-world forcing of complex systems is usually a function that is hard or even barely possible to shape. For instance, targeting a specific rate of global warming to avoid tipping might not be feasible.
- As illustrated in Fig. 4, the presence of noise might induce a non-zero tipping probability close to the separation boundary.

In this work, we have shown that NMRT does not require a high degree of complexity, as it arises in two low-dimensional, non-chaotic, deterministic, oscillatory systems displaying equilibrium points. For the present examples, this counter-intuitive behaviour is due to the peak synchronisation of oscillatory responses, leading to comparatively large magnitudes in the total response. Even in the presence of noise, peak-synchronised NMRT might still be recognisable through Monte-Carlo simulations. The present analysis would have been computationally expensive to drive on models of higher complexity and even partly impossible due to nonlinearities prohibiting the use of the superposition principle. As research on tipping points increasingly accounts for the effect of rate, the impact of NMRT on the results should be taken into account.

## Supplementary Information

Supplementary Information.

## Data Availability

The source code used to generate all the results and the figures is available at https://github.com/JanJereczek/nlo-rtipping. It has been coded in Julia^18^, with major help of the Makie^19^ and DifferentialEquations^20^ packages. The repository was generated with DrWatson^21^, allowing any Julia-user to reproduce the results with a minimal number of commands.
