# Supplementary material for: Time-scale synchronisation of oscillatory responses can lead to non-monotonous R-tipping
Source: Sci Rep. 2023 Feb 6;13:2104. doi: 10.1038/s41598-023-28771-1 (PMC9902488; doi:10.1038/s41598-023-28771-1)
Supplement: Supplementary file 1 — Supplementary Information. [file 41598_2023_28771_MOESM1_ESM.pdf]

Supplementary material

Parameters of PLO

	m [kg]	c_1 [kg s^{-2}]	c_2 [kg s^{-2}]	d [kg s^{-1}]	g [m s^{-2}]	x_T [m]
low-frequency case	100	50	50	20	10	1.5
high-frequency case	1	50	50	varied	10	1.5

Tab. 1: Numerical values of the PLO parameters.

Supplementary plots

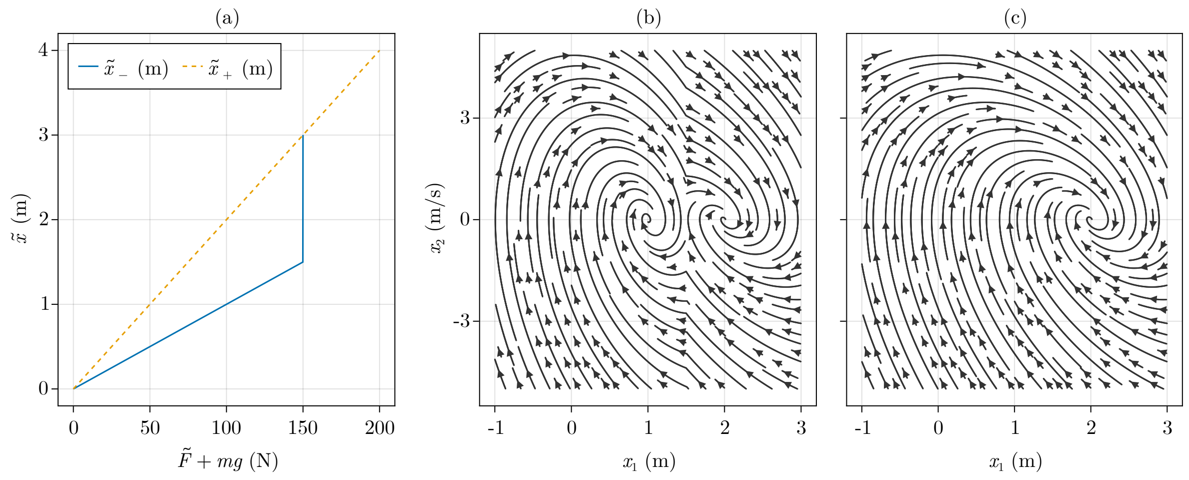

Fig. 1: (a) Bifurcation branches of the PLO for the cases where Spring 2 did not snap ($\tilde{x}_-$) and snapped ($\tilde{x}_+$). The vertical line at $\tilde{F} + mg = 150\,\mathrm{N}$ represents the snapping itself. The two further panels depict the autonomous phase space of the PLO depending on whether the position is iniatiliased as (b) $x_1 < x_T$ or (c) $x_1 \geq x_T$.

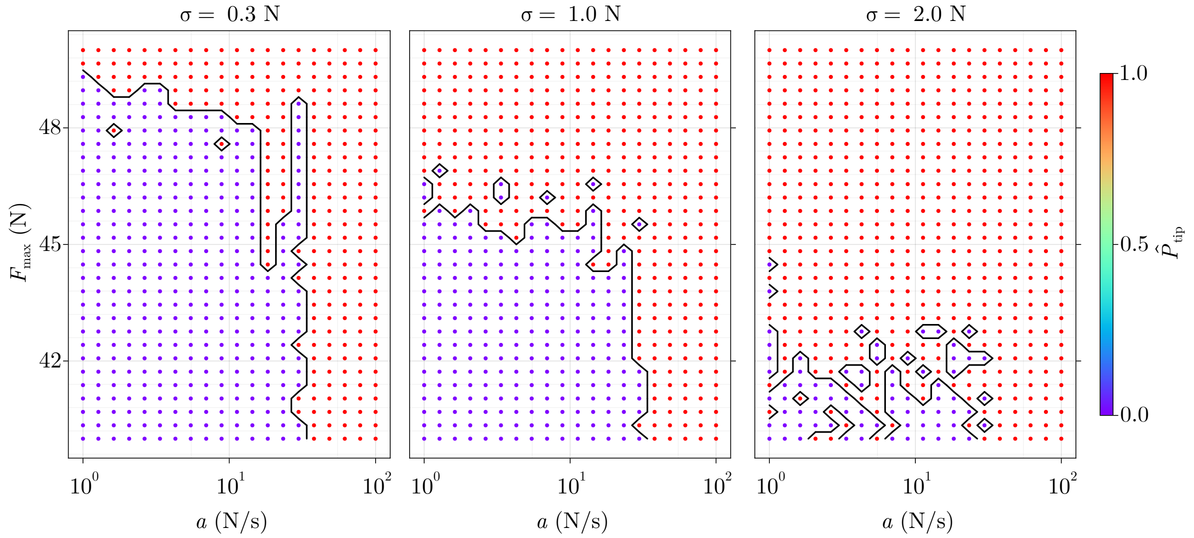

Fig. 2: Tipping pattern represented in the ramp-parameter space for single realisations of $\sigma = \{0.3, 0.1, 2\}$ N and fixed $\Delta x_1 = 0.6$ m. The black line represents the separation function. Whereas the Monte-Carlo experiments on the PLO showed that the structure of the separation function is not altered by the presence of noise (see Fig. 4), single realisations might however change it substantially.

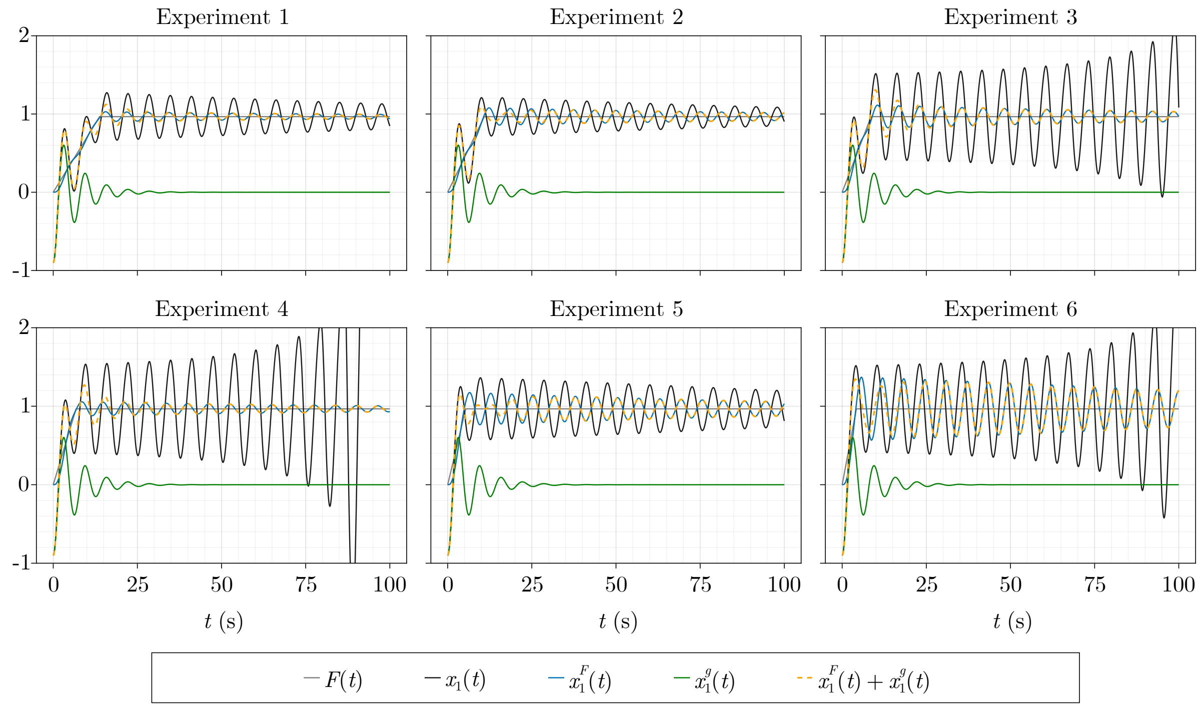

Fig. 3: Transient solutions of the points enclosed by the purple rectangle in Figure 5. Here, the superposition $x_1^F(t)+x_1^g(t)$ does not yield $x_1(t)$ because the VPO is not piecewise-linear. However, tipping is observed whenever the response to forcing $x^F(t)$ is in phase with the one to the initial perturbation $x^g(t)$, i.e. when the sum of both yields higher magnitudes.
